# Mucosal‐associated invariant T cells in autoimmunity, immune‐mediated diseases and airways disease

**DOI:** 10.1111/imm.12582

**Published:** 2016-02-09

**Authors:** Timothy S. C. Hinks

**Affiliations:** ^1^ Department for Microbiology and ImmunologyThe Peter Doherty Institute for Infection and ImmunityUniversity of MelbourneMelbourneVictoriaAustralia; ^2^ Clinical and Experimental SciencesUniversity of Southampton Faculty of MedicineSir Henry Wellcome LaboratoriesSouthampton University HospitalSouthamptonUK; ^3^ NIHR Southampton Respiratory Biomedical Research UnitSouthampton University HospitalSouthamptonUK

**Keywords:** autoimmunity, inflammation, lung, mucosal, T cells

## Abstract

Mucosal‐associated invariant T (MAIT) cells are a novel class of innate‐like T cells, expressing a semi‐invariant T‐cell receptor (TCR) and able to recognize small molecules presented on the non‐polymorphic MHC‐related protein 1. Their intrinsic effector‐memory phenotype, enabling secretion of pro‐inflammatory cytokines, and their relative abundance in humans imply a significant potential to contribute to autoimmune processes. However, as MAIT cells were unknown until recently and specific immunological tools were unavailable, little is known of their roles in disease. Here I review observations from clinical studies and animal models of autoimmune and immune‐mediated diseases including the roles of MAIT cells in systemic lupus erythematosus, rheumatoid arthritis, multiple sclerosis, inflammatory bowel disease and airways diseases. MAIT cell deficiencies are frequently observed in peripheral blood, and at sites of disease such as the airways in asthma. However, MAIT cells have a specific sensitivity to suppression by therapeutic corticosteroids that may confound many of these observations, as may the tendency of the surface marker CD161 to activation‐induced down‐regulation. Nonetheless, the dependence on bacteria for the development of MAIT cells suggests a potentially important protective role linking the influences of early life microbial exposures and subsequent development of autoimmunity. Conversely, MAIT cells could contribute to chronic inflammation either through TCR‐independent activation, or potentially by TCR recognition of as yet undiscovered ligands. Future research will be greatly facilitated by the immunological tools that are now available, including murine genetic models and human and murine specific tetramers.

**Abbreviations**
5‐OP‐RU: 5‐(2‐oxopropylideneamino)‐6‐d‐ribityllumazine
CIA: collagen‐induced arthritis
COPD: chronic obstructive pulmonary disease
EAE: experimental autoimmune encephalomyelitis
HLA: human leucocyte antigen
ICS: inhaled corticosteroids
IFN: interferon
IL: interleukin
iNKT: invariant natural killer T
MAIT: mucosal‐associated invariant T
MR1: MHC‐related protein 1
MS: multiple sclerosis
PLZF: promyelocytic leukaemia zinc finger protein
RhA: rheumatoid arthritis
SLE: systemic lupus erythematosus
TCR: T‐cell receptor
Th17: T helper type 17
TNF: tissue necrosis factor
XIAP: X‐linked inhibitor of apoptosis
XLP: X‐linked lymphoproliferative syndrome

## Introduction

Mucosal‐associated invariant T (MAIT) cells are a subset of innate‐like T lymphocytes first described in 1999,^1^ which are abundant in humans and can rapidly express a range of pro‐inflammatory cytokines. Although MAIT cells express an *αβ* T‐cell receptor (TCR) they differ from conventional T cells in that this receptor has a limited TCR diversity, mostly comprising a semi‐invariant TCR‐*α* chain associated with a limited repertoire of TCR‐*β* chains (Box 1). Furthermore MAIT cells are restricted not by MHC, but by the non‐polymorphic class 1b antigen‐presenting molecule MHC‐related protein 1 (MR1).^2^, ^3^ Ligands for MAIT cells remained elusive until the recent demonstration by Kjer‐Nielsen *et al*. that MR1 presented non‐protein antigens, which include precursors and derivatives from highly conserved biosynthetic pathways of riboflavin and folic acid metabolism in bacteria, mycobacteria and yeast.^4^, ^5^, ^6^ To date, only these limited classes of ligands – most importantly the naturally occurring activating ligand 5‐(2‐oxopropylideneamino)‐6‐*d*‐ribityllumazine (5‐OP‐RU)^7^ – have been identified for MR1 and it remains to be seen whether other classes of ligand exist.

Box 1 **MAIT cell TCR–MR1 recognition**

As with other innate‐like lymphocytes, MAIT cells express a semi‐invariant TCR. Most MAIT cell TCRs comprise a semi‐invariant TCR‐*α* chain – usually TRAV1‐2‐TRAJ33 (V*α*7.2^−^ J*α*33 in humans, V*α*19^−^ J*α*33 in mice),^90^ although in humans some MAIT cells also use TRAV1‐2‐TRAJ12 or TRAV1‐2‐TRAJ20^22^) – predominantly associated with the *β*‐chains TRBV20 (V*β*2) or TRBV6 (V*β*13) in humans^1^ and TRBV19 (V*β*6) or TRBV13 (V*β*8) in mice.^1^, ^22^, ^23^ These preferential TCR rearrangements arise partly through a process of ‘convergent recombination’^91^ whereby much of the antigen specificity of MAIT cells is essentially germline‐encoded. This feature, alongside the striking evolutionary conservation of MR1 across over 150 million years of mammalian evolution,^3^, ^92^, ^93^, ^94^, ^95^ imply a strong evolutionary pressure maintaining the MAIT cell repertoire, and hence some indispensible role in host defence. This limited diversity of MAIT‐TCR, and the abundance of MAIT cells in humans means that, early in an immune response MAIT cells may markedly outnumber responses from conventional peptide‐specific *αβ* T cells.^14^ Abbreviations: MAIT, mucosal‐associated invariant T; MR1, MHC‐related protein 1; TCR, T‐cell receptor; TRAV, TCR‐*α* chain variable region; TRVB, TCR‐*β* chain variable region.

**Figure 1:**
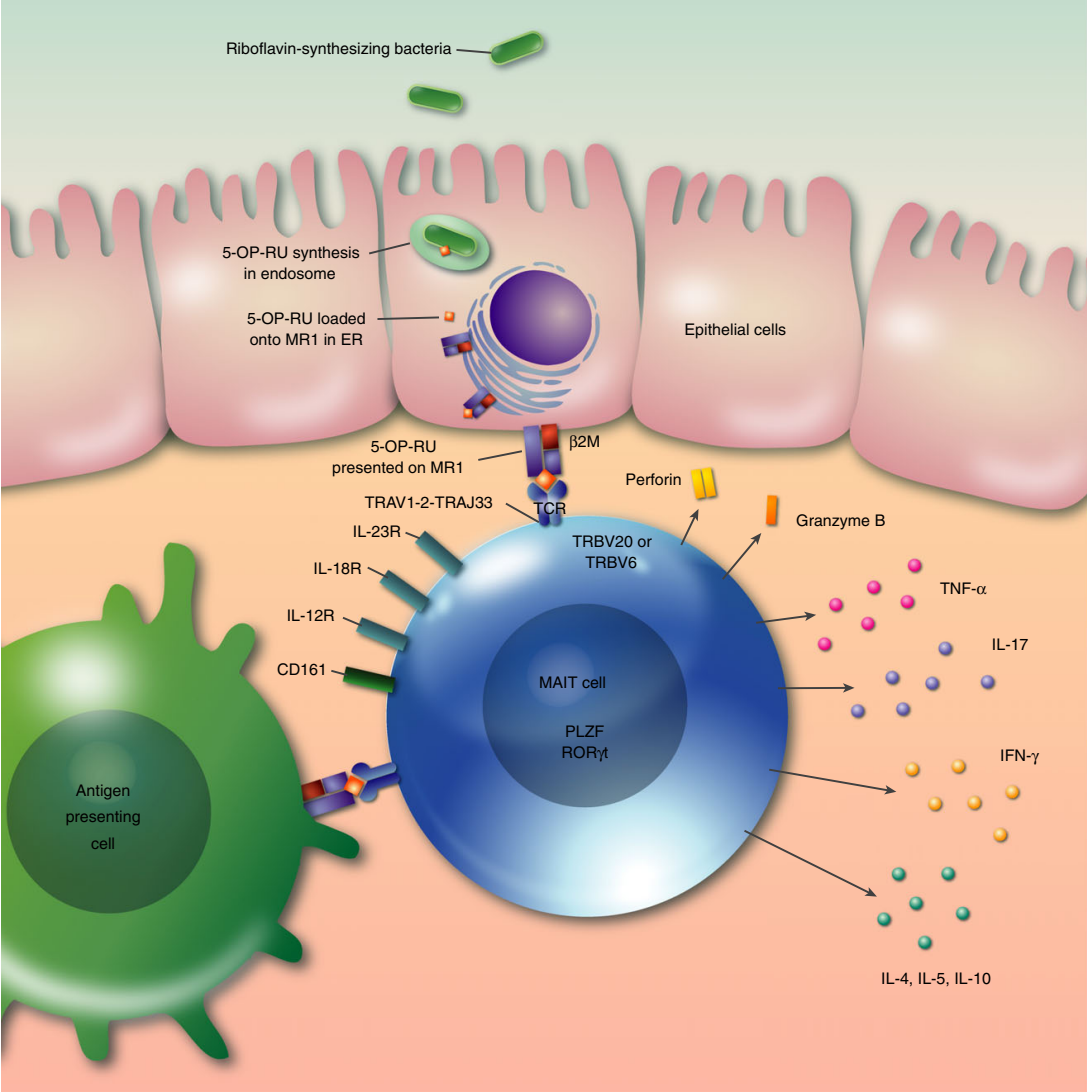
Over view of human MAIT cell biology. Invasive bacteria capable of synthesizing riboflavin enter epithelial cells. Intermediate products of riboflavin synthesis such as 5‐OP‐RU synthesized in the endosome, or potentially delivered by endocytosis, are loaded onto MR1 in the endoplasmic reticulum and transported to the cell surface via the Golgi apparatus. At the surface of infected cells, or antigen‐presenting cells, ligand is presented to the MAIT cell invariant T‐cell receptor, which is predominantly composed of TRAV1‐2‐TRAJ33 *α* chains assembled with TRBV20 or TRBV6 chains. Activation leads to release of perforin and granzyme B, which may directly lyse infected cells, and pro‐inflammatory cytokines including TNF‐*α*, IFN‐*γ*, IL‐17 as well as other cytokines, which may include the type 2 cytokines IL‐4, IL‐5 or the immunoregulatory cytokine IL‐10. MAIT cells typically express the innate lymphocyte transcription factors PLZF and ROR *γ*t and the surface molecules CD161, IL‐12R, IL‐18R and IL‐23R. Abbreviations: 5‐OP‐RU, 5‐(2‐oxopropylideneamino)‐6‐*d*‐ribityllumazine; *β*2M, *β*_2_‐microglobulin; CD, cluster of differentiation; ER, endoplasmic reticulum; IFN, interferon; IL, interleukin; MAIT, mucosal‐associated invariant T; MR1, MHC‐related protein 1; PLZF promyelocytic leukaemia zinc finger protein; ROR *γ*t, retinoic acid receptor‐related orphan receptor *γ* t; TCR, T‐cell receptor; TNF‐*α*, tumour necrosis factor‐*α*; TRAV, TCR‐*α* chain variable region; TRVB, TCR‐*β* chain variable region.

Currently, although there is a growing understanding of the role of MAIT cells in host protection from intracellular pathogens^8^, ^9^, ^10^, ^11^, ^12^, ^13^ (Fig. 1), very little is known concerning the roles that these cells play in disease. Several features suggest potential relevance to immune‐mediated pathology. MAIT cells display an intrinsic effector‐memory phenotype – i.e. without the need for prior clonal expansion^14^ – typically CD45RA^−^ CD45RO^+^ CD95^Hi^CD62L^Lo^ CD44^Hi^ 2, 15, 16, 17 – and can rapidly secrete a range of pro‐inflammatory cytokines including tissue necrosis factor‐*α* (TNF‐*α*), interleukin‐17 (IL‐17) and interferon‐*γ* (IFN‐*γ*), and also the type 2 cytokine IL‐4 on TCR ligation.^15^, ^17^ MAIT cells share some similarities with invariant natural killer T (iNKT) cells, which are implicated in many autoimmune conditions,^18^, ^19^ including expression of a semi‐invariant TCR, restriction by non‐classical MHC molecules, and expression of the transcription factor promyelocytic leukaemia zinc finger protein (PLZF)^20^ although many differences exist, notably the nature of the ligands and the restriction molecule. Other significant features are the remarkable abundance of MAIT cells, which comprise approximately 5% of T cells in peripheral blood^11^, ^17^ and 20–40% of liver T cells in humans,^15^, ^21^ and their wide tissue distribution in blood, mucosal tissues, liver and joints.^15^, ^19^, ^22^, ^23^, ^24^, ^25^ A peculiarity of MAIT cell biology is that although MR1 expression is ubiquitous,^3^, ^26^ it is normally found only at very low levels on the cell surface.^26^, ^27^, ^28^ This has led to speculation that if other classes of MAIT cell ligand exist, they might include autologous molecules whose presentation at the cell surface occurs only when MR1 surface expression is up‐regulated as a signal of cell stress,^2^, ^27^, ^29^ as occurs with other class 1b molecules.^14^, ^30^ Inappropriate triggering of such a system would be expected to lead to immune pathology.

Nonetheless, to date, data regarding MAIT cells in immune‐mediated disease are scant, at least partly because MAIT cells were unknown until recently and specific immunological tools, such as relevant antibodies, transgenic models^16^, ^24^, ^31^ and specific tetramers for humans^7^, ^22^ and mice,^17^ were unavailable. Furthermore, because of the limited diversity of the MAIT TCR and the non‐polymorphic, non‐human leucocyte antigen (HLA) encoded nature of MR1, it is unlikely that there will be pathological autoreactive MAIT cells leading directly to HLA‐associated diseases meeting the stringent definition of a classic autoimmune disease.^32^ Instead, in this paper I will review observational data of MAIT frequency and function in human immune‐mediated diseases, alongside mechanistic data from relevant murine models. These studies are summarized in Table 1. I will then discuss the relevance of corticosteroids and receptor down‐regulation to these studies, the potential of MAIT cells to act as non‐specific effectors of inflammation, and speculate on their relevance in early life origins of immune disease and some potential therapeutic implications.

**Table 1:** Human and murine studies on MAIT cells in immune‐mediated disease

Condition	Human			Mouse			Comment	References
	Blood MAIT frequencies	Tissue MAIT frequencies	Confounding by steroids?	Model	MAIT augmentation	MAIT depletion		
Rheumatological diseases								
Systemic lupus erythematosus	↓ correlating with disease activity		Yes	CIA and CAIA	↑ severity after adoptive MAIT transfer	↓ severity in MR1^−/−^	MAIT cells are likely effector cells	Cho et al.^19^
	↓ IFN‐γ‐secreting MAIT							Chiba et al.^40^
	↑ PD1 expression							
Rheumatoid arthritis	↑ correlating with disease activity	↑ in synovium in disease	Yes					
Multiple sclerosis	↓ especially in relapse, improving with remission	Present in inflammatory lesions in MS and CIDP	Yes	EAE	↓ incidence and severity in Vα19i TG mice	↑ severity in MR1^−/−^, with ↑ inflammatory cytokines and ↓ IL‐10	Murine data suggest MAIT cells protective, but data confounded by ‘MAIT‐like’ cells	Illes et al.^47^
					↓ pathology by adoptive transfer of Vα19i cells			Miyazaki et al.*,* 49 Croxford et al.^50^
Inflammatory bowel disease								
Crohn's	↓ with MAIT cell activation	↑ in ileal tissue in disease	Yes	TNBS	↑ severity after adoptive MAIT	Murine data confounded by non‐ MAIT conventional T cells		Serriari et al.,^29^ Hiejima et al.^54^ Hinks^55^
Ulcerative colitis	↓		Yes					
Coeliac disease	↓	↓ in lamina propria	No					Dunne et al.^59^
Airway diseases								
Asthma	↓ if taking ICS	↓ in sputum and bronchial biopsies if taking ICS	Yes				Deficiency might contribute to increased risks of pneumonia. Potential protective role of MAIT cells in early life	Hinks et al.,^44^ Hinks et al.,^45^ Unpublished observations
COPD	↓ if taking ICS	↓ in bronchial biopsies if taking ICS	Yes					
Obesity	↓ and normalises after bariatric surgery	↑ in adipose tissue in obesity					Associated with insulin resistance	Magalhaes et al.,^82^ Carolan et al.^83^
		↑ IL‐17/IL‐10 profile						

CAIA, collagen antibody‐induced arthritis; CIA, collagen‐induced arthritis; CIDP, chronic inflammatory demyelinating polyneuropathy; COPD, chronic obstructive pulmonary disease; DN, double negative; EAE, experimental autoimmune encephalomyelitis; ICS, inhaled corticosteroids; IFN, interferon; MAIT, mucosal‐associated invariant T; MS, multiple sclerosis; PD‐1, programmed cell death protein 1; TG, transgenic; TNBS, 2,4,6‐trinitrobenzenesulphonic acid.

It is important to compare and contrast the biology of MAIT cells in humans and in mice, as differences may explain some discrepancies between human disease and animal models. In both species MAIT cells express orthologous TCR‐*α* chains, are MR1 restricted and recognize the same antigen. In both species MAIT cells express the master transcription factor PLZF and signature surface markers including CD127 (IL7R*α*), CD218 (IL18R*α*), CCR9 and CCR6.^17^ However, by contrast, MAIT cells are at least 10‐fold less abundant in mice than in humans.^17^, ^27^ Although some other previously reported species differences may have been artefacts of a specific transgenic murine model,^17^ other differences include a more limited expression of CD161 in mice^17^, ^24^ and, of relevance to this review, a difference in IL‐17 production. Interleukin‐17 is abundantly produced by both mouse and human MAIT cells in response to mitogen, but in humans IL‐17 is not produced in response to TCR triggering.^17^, ^25^, ^33^ In general each section of this review discusses human data from clinical studies first, before addressing murine data where relevant.

## Observations from specific diseases

### Systemic lupus erythematosus and rheumatoid arthritis

Systemic lupus erythematosus (SLE), an archetypal multi‐system autoimmune disease, is considered a multi‐factorial disorder with evidence of genetic susceptibility, environmental triggers including infections, and dysregulation of innate and adaptive immunity.^34^, ^35^ The pro‐inflammatory cytokine IL‐17 is associated with SLE and correlated with disease activity,^36^, ^37^ whereas IL‐17‐producing T helper type 17 (Th17) cells are involved in driving local inflammation at sites of disease.^38^, ^39^ MAIT cells are potent producers of IL‐17 and due to their similar cytometric profile may in fact have constituted many of the ‘Th17’ populations measured in these studies.^40^ Local cytokine production by MAIT cells within tissues could contribute to lowering the quantitative threshold of immune signalling, a process believed to interact with genetic predispositions to contribute to the initial pathogenic processes in SLE.^35^ Furthermore, several non‐MHC loci contribute to genetic predisposition to SLE,^35^ so potentially polymorphisms in MR1 or other MAIT‐cell related genes could contribute to such predispositions, although to date these have not been described.

An important feature of MAIT cells is that they can be stimulated by cytokines without the need for specific TCR stimulation. Hence, human and murine MAIT cells can produce IFN‐*γ* 41 or IL‐17^19^, ^40^ in response to IL‐12, and IL‐18 or IL‐23, respectively. Given their abundance this may constitute the most important effect of MAIT cells in autoimmunity as they non‐specifically amplify pro‐inflammatory signals within the cytokine milieu, enhancing inflammation in both rheumatological conditions like SLE and other diseases.

Cho *et al*.^19^ report a reduced frequency of human MAIT cells in peripheral blood in patients with both SLE and rheumatoid arthritis (RhA), particularly in the CD8^+^ and double‐negative subsets, which correlated with disease activity scores in both conditions. Furthermore, frequencies of MAIT cells secreting IFN‐*γ* (though not IL‐17 or IL‐4) were reduced in peripheral blood in SLE, with a similar trend in RhA, attributable to a defect in Ca^2+^/calcineurin/nuclear factor of activated T cells 1 signalling. This study reported increased expression of the co‐inhibitory molecule programmed cell death protein 1, perhaps a consequence of chronic MAIT‐cell activation leading to T‐cell exhaustion. At the site of disease MAIT cells were increased in synovial tissue in human RhA,^19^ and so might contribute to maturation and cross‐differentiation of T cells within the tissue microenvironment.^35^ These findings suggest possible recruitment of MAIT cells to sites of disease.

It should be noted that in this study, as with all the human clinical studies described in this review, MAIT cells were defined by surface phenotype (TCR V*α*7.2^+^ CD161^Hi^) but not using MAIT cell‐specific tetramers, and are therefore susceptible to confounding by CD161 down‐regulation, as occurs with MAIT cell stimulation,^22^, ^42^, ^43^ which may lead to a failure to detect MAIT cells,^42^ which are truly present in the clinical samples. Of greater significance here is potential confounding by corticosteroid therapy. As will be discussed later, MAIT cell frequencies are very sensitive to therapeutic corticosteroids, which cause a rapid decrease in MAIT cell frequencies.^44^, ^45^ Indeed Cho *et al*.^19^ observed decreased MAIT cell frequencies only in patients with SLE or RhA, of which 90% were receiving corticosteroids, but no deficiency in two other autoimmune diseases also studied – ankylosing spondylitis and Behçet's disease – in which no patients were receiving steroids. Although their regression analysis did not detect steroid use as a significant coefficient, this may have been apparent if more non‐steroid‐treated patients were available for analysis or steroid dose had been analysed as a continuous variable. Irrespective of the effects of therapy, in such observational studies it is hard to determine causality: whether changes in MAIT cell frequency are a primary effect or merely reflect a response to cytokines produced by immune dysregulation.^35^

By contrast with this observed MAIT cell deficiency, a pathogenic role is implied by data from a murine collagen‐induced arthritis (CIA) model. Chiba *et al*.^40^ found that MR1^−/−^ mice, which lack MAIT cells, had reduced severity of CIA and of collagen antibody‐induced arthritis, whereas CIA was recapitulated by reconstitution with adoptively transferred MAIT cells. As antigen‐specific responses of other T and B cells were unaffected by the presence of MR1, MAIT cells seem to be acting as effectors rather than initiators of inflammation in this model.

### Multiple sclerosis

Autoimmune T‐cell responses by Th1 and Th17 cells against components of myelin play an important pathogenic role in multiple sclerosis (MS) and other demyelinating conditions.^46^ In humans, MAIT cells were first reported in inflammatory lesions in MS, chronic inflammatory demyelinating polyneuropathy^47^ and neurological tumours^48^ using PCR for the canonical V*α*7.2^−^ J*α*33 TCR. It will be important to verify the specificity of these cells because non‐MAIT cells can express V*α*7.2. More recently, using cytometry, frequencies of V*α*7.2^+^ CD161^+^ MAIT cells have been shown to be decreased in peripheral blood of MS patients, particularly during disease relapse, compared with remission,^49^ and MAIT cell frequencies increased in paired samples 2–3 months after cessation of corticosteroid therapy. MAIT cells expressed CCR5 and CCR6, necessary for central nervous system invasion, suggesting that they might preferentially migrate to central nervous system lesions, and peripheral MAIT frequencies correlated positively with frequencies of iNKT and natural killer cells. This study sampled peripheral blood only and again these findings, particularly the paired analysis of relapse and post‐relapse remission, will be confounded by the use of corticosteroids, which are central to the management of MS relapse, and which may have long‐lasting suppressive effects on MAIT cell frequencies.

Nonetheless, elegant murine data from experimental autoimmune encephalomyelitis (EAE), a murine model of MS, supports a potentially protective role of MAIT cells in neuro‐inflammation. As there is no specific antibody for the canonical V*α*19i TCR chain in mice, and wild‐type mice have very few MR1‐restricted T cells, Croxford *et al*.^50^ used transgenic mice over‐expressing V*α*19i TCR, in which T cells that express the natural killer marker NK1.1^+^ are enriched for MAIT cells expressing V*α*19^−^ J*α*33 TCR with V*β*6 or V*β*8 TCR chains. Incidence and severity of EAE were decreased in V*α*19i transgenic mice by clinical and histological scores, and spinal cord lesions had less infiltration by monocytes and CD4^+^ T cells, with less demyelination. Similarly, EAE was ameliorated in wild‐type mice by adoptive transfer of V*α*19i T cells. Lymph node T cells from these mice produced less pro‐inflammatory cytokines and more IL‐10. Conversely, MR1^−/−^ mice, which lack MAIT cells, showed more severe EAE, with earlier onset, and more T‐cell proliferation and pro‐inflammatory cytokine production, with less IL‐10 production. In co‐culture studies V*α*19i T cells induced IL‐10 from iNKT cells and conventional T cells, but most potently from CD19^+^ B cells, and this was, at least partly, by an MR1‐independent mechanism through inducible T‐cell co‐stimulator–B7 related protein‐1 interactions. One important limitation of the methodology used in this and other^16^, ^24^, ^31^ studies is that the V*α*19i population includes many MHC‐restricted ‘MAIT‐like’ cells that do not stain with the specific MR1 tetramer or express signature innate cell transcription factors like PLZF.^17^, ^22^ Together these findings suggest that MAIT cells are protective against autoimmune demyelinating inflammation by a suppressive effect on other B and T cells, particularly by enhancing IL‐10 secretion and inhibiting Th1 cytokines.

### Inflammatory bowel disease and enteropathies

The MAIT cells were shown early on to be present in the human gut lamina propria,^1^, ^2^ to express the gut homing integrin *α*_4_/*β*_7_, and have been considered to be preferentially located in mucosal tissue.^27^ Moreover an important role in gastrointestinal mucosal immunity is suggested by the dependence on commensal gut flora for their development in mice.^2^, ^16^ It is therefore likely that MAIT cells play a role in disorders of gastrointestinal mucosal immunity.

Chronic haemorrhagic colitis is a common feature of X‐linked lymphoproliferative syndrome (XLP) type 2.^20^, ^51^ XLP‐2 is a rare human genetic immunodeficiency caused by mutations in the X‐linked inhibitor of apoptosis (XIAP) gene, leading to effects including increased apoptosis of iNKT and MAIT cells.^20^, ^51^ XLP was therefore the first identified inherited immunodeficiency associated with iNKT or MAIT cell defects, with a 10‐fold decrease in peripheral blood MAIT cell frequencies.^20^ Colitis occurs in 17% of patients with XLP‐2 and resembles a severe form of inflammatory bowel disease, with accumulations of activated T cells and a mortality of 60%.^51^ Although XIAP deficiency may have pleiotropic effects on the immune system, including hypogammaglobulinaemia, it seems likely that the colitis is a direct consequence of the MAIT cell deficiency.

Crohn's disease is believed to be initiated and driven by CD4^+^ T cells targeting the commensal gut microbiota, and secreting IL‐12, IL‐17, TNF‐*α* and IFN‐*γ*.^29^ Ulcerative colitis is likely to involve similar mechanisms, but also Th2 cells secreting IL‐5 and IL‐13 and CD1d‐restricted type II NKT cells.^52^, ^53^ As with SLE, RhA and MS, in cross‐sectional observational studies there is a deficiency of human MAIT cells in peripheral blood in both Crohn's disease and ulcerative colitis,^29^, ^54^ although at least some of this effect is likely to be attributable to current or recent use of therapeutic corticosteroids, whether topical or systemic.^55^ Other possible factors include selective apoptosis of MAIT cells due to chronic activation‐induced cell death or recruitment to sites of disease. Indeed, consistent with the former possibility, Serriari *et al*.^29^ observed increased expression of the activation markers Ki67, NKG2D and BTLA receptor in human blood MAIT cells, which also had enhanced IL‐17 production. Consistent with the latter suggestion these authors also observed greater than fourfold accumulation of CD3^+^ IL18R*α* ^+^ V*α*7.2^+^ MAIT cells in biopsies of inflamed tissue from Crohn's disease ileum compared with biopsies from healthy sections.^29^

It is not clear whether MAIT cells actively contribute to this inflammation. One report suggests that adoptive MAIT transfer is protective in a murine 2,4,6‐trinitrobenzenesulphonic acid colitis model, but this study depended on only an in‐house antibody to J*α*33 TCR, and therefore is confounded by non‐MAIT conventional T cells.^56^ It seems more likely from their known cytokine secretion profile that they would contribute to inflammation at sites of disease. Given the known role of MAIT cells in responding to riboflavin‐synthesizing microbes it is also possible that MAIT cell recruitment and activation is a consequence of the dysbiosis of the gastrointestinal microbiome that occurs in inflammatory bowel disease.^57^, ^58^ By contrast, human lamina propria and blood V*α*7.2^+^ CD161^+^ MAIT cells are deficient in untreated coeliac disease,^59^ another important autoimmune enteropathy.^60^ Coeliac disease is driven by exposure to dietary gluten in genetically predisposed people, but it can be associated with compromised mucosal barrier function and bacterial overgrowth and therefore has the potential for activation‐induced MAIT cell death.^59^

### Airways diseases

**Figure 2:**
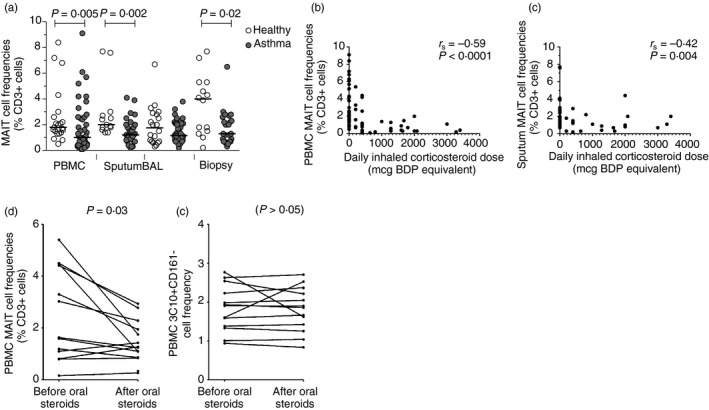
MAIT cells are deficient in asthma and this is related to corticosteroids. Frequencies of V*α*7.2^+^ CD161^+^ (MAIT) cells as a proportion of total live CD3^+^ T cells (a) in peripheral blood, sputum, bronchoalveolar‐lavage (BAL) and bronchial biopsies in health and asthma. Horizontal lines represent medians. *P*‐values are for unpaired *t*‐tests on log‐transformed data. Frequencies of MAIT cells in (b) peripheral blood and (c) induced sputum correlate inversely with the dose of inhaled corticosteroid. Frequencies of MAIT cells (d), but not conventional T cells (e), were suppressed by 7 days of treatment with 20 mg oral prednisolone in 12 subjects with moderate asthma (23% fall in median frequency, *P*‐values are for paired *t*‐tests). Abbreviations: BDP, beclomethasone dipropionate; *r*_*s,*_ Spearman's correlation coefficient. Figure adapted from Elsevier Hinks.^44^

The role that innate‐like lymphocytes play in airways disease has evoked some controversy.^61^, ^62^, ^63^ Whereas iNKT cells are implicated in murine models of allergic airways disease, they are rare in the human airways;^62^ in comparison, MAIT cells are fivefold to tenfold more abundant in humans than in mice.^27^ Asthma is characterized by airways inflammation, classically associated with activated allergen‐specific Th2 cells^64^ secreting IL‐4, IL‐5 and IL‐13, which orchestrate the function of eosinophils, mast cells and B cells.^44^, ^65^ There is increasing recognition of disease heterogeneity and some genetic^66^, ^67^ and murine^68^, ^69^ data implicating IL‐17 in certain phenotypes of asthma. We conducted a large‐scale bronchoscopy‐based study of 60 patients spanning the spectrum of asthma severity and phenotypes and 24 healthy controls analysing multiple T‐cell subsets in the context of deep clinical and immunological phenotyping.^44^ We found no evidence of dysregulation of IL‐17‐secreting CD4^+^ Th17 cells, but observed a striking deficiency of V*α*7.2^+^ CD161^+^ T cells in blood, sputum and bronchial biopsy samples^44^, ^45^ (Fig. 2). This deficiency correlated with disease severity in both blood and sputum and was associated with poor asthma control, poor lung function, longer disease duration and dose of inhaled corticosteroids (ICS). We conducted two open‐label clinical trials of ICS and oral corticosteroids. There was no change in blood or sputum MAIT cell frequencies with a 7‐day moderate dose ICS (twice‐daily Qvar 200 μg), but there was a significant 23% decrease in peripheral blood MAIT cell frequencies (*P* = 0·03) with just 7 days of systemic corticosteroids (once‐daily prednisolone 20 mg). This effect was specific to MAIT cells; it was not observed with V*α*7.2 TCR‐expressing conventional T cells.^44^, ^45^

We have since observed a similar deficiency of blood and bronchial biopsy MAIT cells in chronic obstructive pulmonary disease, which is apparent only in individuals receiving therapeutic ICS (Hinks *et al*., unpublished observations). Together these findings have important implications both for the management of airways diseases and for other conditions. It is remarkable that a systemic deficiency in peripheral blood MAIT cells can be induced even by the small doses of corticosteroid that are absorbed systemically after administration of low‐dose ICS,^70^ such as the patients with moderate asthma in our study who received a median 400 μg beclomethasone dipropionate daily equivalent. Therefore, it seems likely that much of the observational human data from other diseases discussed in this review are attributable, at least in part, to current or recent corticosteroid therapy.

What might be the consequences of these therapy‐induced deficiencies? MAIT cells are likely to contribute significantly to protection from pulmonary infections. Indeed 27% of individuals with XLP‐2 suffer from recurrent respiratory infections,^51^ although hypogammaglobulinaemia will also be relevant. Steroid‐induced suppression of airway MAIT cells might underlie the increased risk of pneumonia and invasive pneumococcal disease associated with severe asthma^71^, ^72^ and increased pneumonia risk in subjects with chronic obstructive pulmonary disease receiving inhaled fluticasone,^73^, ^74^ or contribute to chronic airway colonization with riboflavin‐synthesizing pathogens, notably *Haemophilus influenzae* in asthma and chronic obstructive pulmonary disease.^75^

Although MAIT cells are likely to protect against chronic bacterial infections of the airways, they may also play a role in the pathogenesis of allergic disease. Most MAIT cells secrete type 1 cytokines but some clones can produce type 2 cytokines.^17^, ^23^, ^50^ It is possible that in early life during initiating events in the development of allergic and autoimmune disease, such conditions are associated with a skewing towards a type 2 cytokine‐secreting profile in MAIT cells, as occurs with other T‐cell subsets,^44^, ^64^ and may occur early in life. Exposure to microbes during early childhood is associated with protection from immune‐mediated diseases.^76^, ^77^, ^78^ One mechanism may be persistent effects on numbers and function of innate lymphocytes, such as the accumulation of iNKT cells, which occurs in the lamina propria and lungs of germ‐free mice, resulting in increased morbidity in models of inflammatory bowel disease and allergic airways inflammation.^77^, ^79^ As commensal flora are absolutely required for MAIT cell development, differences in early life exposures would be expected to also impose long‐lasting effects on MAIT cells, which could well play an important role in the tendency towards the initial development of atopy, allergy and autoimmunity.

### Obesity and type II diabetes

There is growing recognition of the importance of immune components to a wider range of diseases than previously considered, and this produces a potentially broader relevance of MAIT cells. For example, adipose tissue is immunologically active and obesity causes a sterile inflammation^80^ in which iNKT may play an immunoregulatory role.^81^, ^82^ MAIT cells may contribute to this inflammation because in human obesity they are enriched in adipose tissue compared with blood, and have a shift towards higher IL‐17 production and reduced IL‐10 secretion.^82^, ^83^ In peripheral blood, MAIT cell frequencies are reduced in obesity and in type 2 diabetes, even below the limit of detection in some severely obese individuals,^82^ and display a more activated phenotype, with up‐regulated CD25 and IL‐17 production. Both these studies found correlations between MAIT frequencies and insulin resistance, perhaps related to IL‐17 production, and Magalhaes *et al*. observed some normalization of MAIT cell phenotype and number after bariatric surgery. Hence MAIT cells may act as effectors contributing to the pathology of obesity‐associated insulin resistance. Furthermore, as with inflammatory bowel disease, obesity is associated with dysbiosis,^84^ which may therefore affect gastrointestinal MAIT cell functions and contribute to the poorly understood mechanistic links between the microbiome and obesity.

## Perspective and future directions

**Figure 3:**
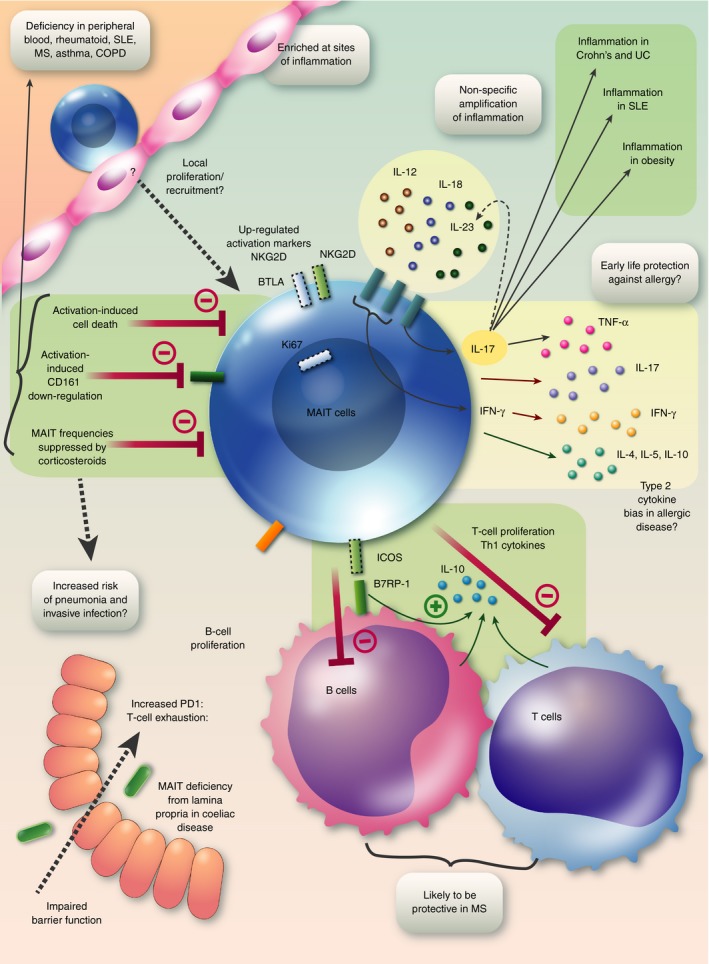
Graphical summary: MAIT cells in immune‐mediated disease. A deficiency of MAIT cells in peripheral blood is probably due to several factors: suppression of MAIT cell frequencies by corticosteroids, activation‐induced cell death and down‐regulation of the popular marker CD161. The consequences of such a deficiency may include increased risk of pneumonia and invasive infections. MAIT cells are enriched at sites of disease, probably as a result of local proliferation and recruitment, and up‐regulate activation markers including NKG2D, BTLA and Ki67. Chronic stimulation may lead to T‐cell exhaustion with PD‐1 up‐regulation. Secretion of pro‐inflammatory cytokines, especially IL‐17 will contribute to inflammation in inflammatory bowel disease, SLE and obesity. Non‐TCR‐mediated secretion of IL‐17 may be driven by IL‐23 and secretion of IFN‐*γ* can be driven by IL‐12 and IL‐18, allowing MAIT cells to amplify inflammatory signals in a non‐specific manner. It is possible that in allergic disease MAIT cells have a bias towards secretion of type 2 cytokines, whereas in early life microbial exposures may drive MAIT cells to produce a type 1 cytokine response protecting against the development of allergic diseases. MAIT cells may be protective in multiple sclerosis by inducing IL‐10 from T cells and particularly B cells by an MR1‐independent mechanism via ICOS–B7RP‐1 interaction. Abbreviations: B7RP‐1, B7 related protein‐1; COPD, chronic obstructive pulmonary disease; ICOS, inducible T cell co‐stimulator; IFN, interferon; IL, interleukin; MAIT, mucosal‐associated invariant T; MR1, MHC‐related protein 1; MS, multiple sclerosis; PD1, programmed cell death protein 1; SLE, systemic lupus erythematosus; TCR, T‐cell receptor; TNF‐*α*, tumour necrosis factor‐*α*; UC, ulcerative colitis.

Several common themes emerge from these studies, summarized in Fig. 3. Although our knowledge of the emerging field of MAIT cell biology is limited, several factors implicate these cells in immune‐mediated diseases, including their pro‐inflammatory profile,^40^ their widespread tissue distribution and effector‐memory phenotype. However, many clinical studies to date are confounded by the effect of therapeutic steroids, and others that use a V*α*7.2CD161^+^ definition of MAIT cells may be confounded by the down‐regulation of CD161 which occurs with chronic activation.^22^, ^85^ Moreover, most clinical data are only observational, making it hard to determine whether aberrations are primary phenomena, or perhaps represent a response to the effects of a disordered cytokine milieu.^35^ Indeed, given their remarkable abundance and the potential for TCR‐independent activation of MAIT cells by cytokines such as IL‐12, IL‐18 and IL‐23, the most important effects of MAIT cells in autoimmunity could be non‐specific amplification of inflammatory cytokines leading to increased IFN‐*γ* 41 and IL‐17.^19^, ^40^ Differences between murine models and human studies may be due to such confounding factors in human studies as well as differences between the species.

The following questions should constitute priorities for future research. What other ligands can MAIT cells recognize? Do MAIT cells display significant reactivity to self‐ligands? How can MAIT cells distinguish between colonization and infection, leading to immune tolerance or inflammation? Can a lack of early‐life MAIT cell stimulation contribute to the development of autoimmunity^35^, ^86^ and allergy,^76^ perhaps through a skewing towards a type 2 cytokine profile, or a deficiency of MAIT cells? Do MAIT cells undergo activation‐induced depletion in autoimmune disease?

The tools necessary to answer these questions now exist, including specific human^22^ and murine^17^ MR‐1 tetramers, antibodies against MR1^87^ and the MAIT cell TCR,^24^ and transgenic mice over‐expressing V*α*19 T cells.^16^, ^24^, ^31^, ^88^ The strong evolutionary conservation of MR1 suggests that these tools will soon reveal some critical immune functions for these enigmatic cells. Furthermore, as the MAIT cell ligands are small molecules, this population could be easily targeted with drugs such as MAIT inhibitory ligands,^89^ making it an exciting time to speculate on the range of clinical applications that will surely emerge.

## Disclosures

The author declares no financial conflicts of interest.
